# Pharmacological Effects of Astragaloside IV: A Review

**DOI:** 10.3390/molecules28166118

**Published:** 2023-08-18

**Authors:** Yutong Liang, Biqiong Chen, Di Liang, Xiaoxiao Quan, Ruolan Gu, Zhiyun Meng, Hui Gan, Zhuona Wu, Yunbo Sun, Shuchen Liu, Guifang Dou

**Affiliations:** 1 Beijing Institute of Radiation Medicine, Beijing 100091, China; liangyutongllg@163.com (Y.L.); liangdi0807@163.com (D.L.); qxxaxxxx@163.com (X.Q.); gurl311@126.com (R.G.); mengzhiyun@vip.163.com (Z.M.); ganh2003@163.com (H.G.); wznphd@126.com (Z.W.); sunyunbo0919@126.com (Y.S.); 2 Institute of Chinese Medicine, Heilongjiang University of Chinese Medicine, Harbin 150040, China; chenbiqiong0501@163.com; 3 Scientific Experimental Center of Guangxi University of Chinese Medicine, Nanning 530200, China

**Keywords:** astragaloside IV, anti-inflammatory, antioxidative, neuroprotective, antifibrotic, antitumor, pharmacological action

## Abstract

Astragaloside IV (AS-IV) is one of the main active components extracted from the Chinese medicinal herb Astragali and serves as a marker for assessing the herb’s quality. AS-IV is a tetracyclic triterpenoid saponin in the form of lanolin ester alcohol and exhibits various biological activities. This review article summarizes the chemical structure of AS-IV, its pharmacological effects, mechanism of action, applications, future prospects, potential weaknesses, and other unexplored biological activities, aiming at an overall analysis. Papers were retrieved from online electronic databases, such as PubMed, Web of Science, and CNKI, and data from studies conducted over the last 10 years on the pharmacological effects of AS—IV as well as its impact were collated. This review focuses on the pharmacological action of AS-IV, such as its anti-inflammatory effect, including suppressing inflammatory factors, increasing T and B lymphocyte proliferation, and inhibiting neutrophil adhesion-associated molecules; antioxidative stress, including scavenging reactive oxygen species, cellular scorching, and regulating mitochondrial gene mutations; neuroprotective effects, antifibrotic effects, and antitumor effects.

## 1. Introduction

**Figure 1:**
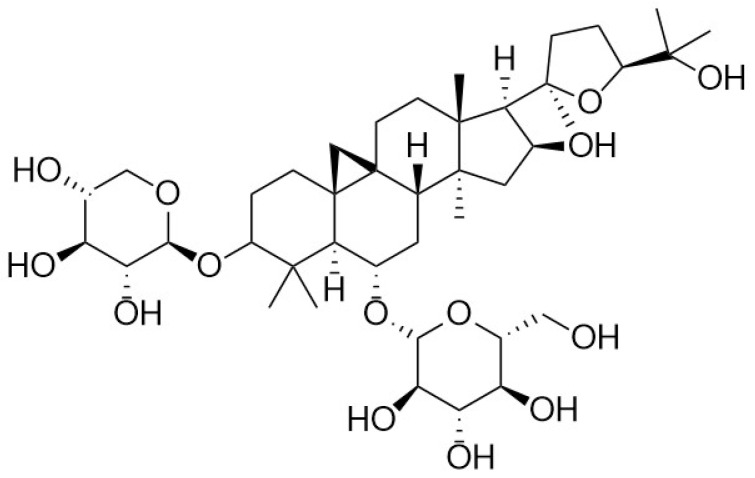
The structural formula of AS-IV.

Polysaccharides and astragalosides are the main active components of the traditional Chinese medicine astragalus. Astragalosides can be categorized into astragalosides I, II, and IV, with astragalosides IV (AS-IV) being the most biologically active compound [1]. AS-IV is a tetracyclic triterpenoid saponin in the form of lanolin ester alcohol and possesses the efficacy of an astragalus polysaccharide with excellent potency. AS-IV has poor water solubility; however, it is readily soluble in methanol, ethanol, and dimethyl sulfoxide. Its molecular formula is C_41_H_68_O_14_, and its molecular weight is 784.97 Da; its structure is shown in Figure 1. In recent years, scientific studies on astragalus methyl glycosides have demonstrated a wide range of pharmacological activities. In particular, AS-IV exhibits various pharmacological effects, including anti-inflammatory, antifibrotic, antioxidative stress, antidiabetic, and cardioprotective effects, which can be triggered by modulating different signaling pathways. AS-IV has been reported to be effective in treating various diseases [2], such as cerebral ischemia/reperfusion lesions, cardiovascular diseases, lung disease, liver cirrhosis, and diabetic nephropathy (DN). In addition, increasing evidence implicates AS-IV in organ fibrosis, the inflammatory response, oxidative stress, and apoptosis. This review provides a systematic overview of the pharmacology, metabolism, and therapeutic molecular mechanisms, as well as the medicinal significance of AS-IV, to provide a reference for future research and applications related to astragalosides.

## 2. Anti-Inflammatory Effects

**Figure 2:**
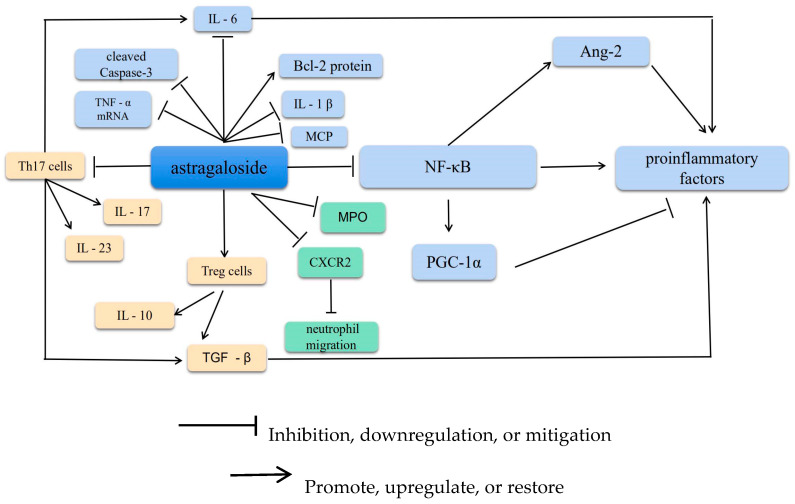
The anti-inflammatory mechanism of AS-IV. Annotation: IL-6: Interleukin 6; IL-10: Interleukin 10; IL-17: Interleukin 17; IL-23: Interleukin 23; Bcl-2 protein: B-cell lymphoma-2; IL-1β: Interleukin-1β; MCP: Monocyte chemotactic protein; TNF-α mRNA; tumor necrosis factor-α; Th17 cells: Helper T-17 cells; TGF-β: Transforming growth factor beta; CXCR2: Chemokine Receptor 2; NF-kB: Nuclear factor-k-gene binding; PGC-1α: Peroxisome proliferator-activated receptor- γ coactivator; Ang-2: Angiopoietin-2.

The inflammatory response is a key feature in the transition from mild to severe and critical illness. The inflammatory response is a reflection of the body’s defense against inflammatory factors. AS-IV exhibits anti-inflammatory effects and has been reported to reduce various types of inflammatory injuries, such as lipopolysaccharide (LPS)-induced organ damage, ischemia–reperfusion injury, allergic diseases, diabetes mellitus and its complications, myocardial injury, and hypertension [3]. These diseases are related to the downregulated gene expression of some inflammation-related factors. AS-IV involves activating transcription factors and downstream inflammatory cytokine release [4]. AS-IV exerts potent antagonistic effects on inflammation via multiple pathways. For example, AS-IV counteracts inflammatory damage by regulating inflammatory factors (interleukin [IL]-1β, tumor necrosis factor [TNF]-α, intercellular adhesion molecule [ICAM], and chemokines), inflammatory mediators (nitrogen oxide [NO]), the nuclear factor kappa-light-chain enhancer of activated B cells (NF-κB) signaling pathway, and apoptosis-related genes. Here, the mechanism of the anti-inflammatory effect of AS-IV has been described and is also depicted in Figure 2.

### 2.1. Suppression of Inflammatory Factors

Increased expression of proinflammatory factors disrupts the homeostasis of the inflammatory microenvironment. The NF-κB protein is central to the inflammatory response. Cell stimulation activates the inhibitor of NF-κB (IκB) and decreases IκB phosphorylation. Furthermore, ubiquitination modifies the IκB subunit of the NF-κB–IκB complex, allowing NF-κB to dissociate, enter the nucleus, and initiate transcription. Reportedly, NF-κB triggers the release of proinflammatory factors, such as IL-6 and TNF-α [5]. In addition, some experiments have demonstrated that astragaloside activates the cholinergic anti-inflammatory pathway by restoring the Th17/Treg balance by impeding CXCR4 to ameliorate COPD, consequently reducing the symptoms in pulmonary embolism rat models [6]. Moreover, AS-IV can inhibit NF-κB–p65 pathway-mediated inflammation in the placenta. Astragaloside has further been shown to attenuate *Streptococcus pneumoniae*-induced inflammatory damage in alveolar epithelial cells by upregulating mRNA and Bcl-2 protein expression; downregulating monocyte chemoattractant protein (MCP) and NF-κB protein expression; and Bax, cleaved caspase-3, IL-1β, IL-6, and TNF-α mRNA expression; and reducing apoptosis [7].

Peroxisome proliferator-activated receptor-γ coactivator-1α (PGC-1α) expression is inhibited in the hippocampus of chronic unpredictable stress or LPS mouse models. Furthermore, astragaloside increases PGC-1α expression, attenuates depression-like behavior, and reduces neuroinflammation by modulating NF-κB signaling. Reducing PGC-1α levels has been reported to reverse the effects of astragaloside on NF-κB signaling and neuroinflammation [5].

The anti-inflammatory properties of astragalus also play a vital role in treating lymphatic vessel growth and lymphedema. In an inflammatory environment, NF-κB is a promoter of Ang-2. Upregulating its expression can therefore augment Ang-2 protein synthesis, ultimately triggering and promoting the growth of inflammatory lymphatic vessels [8].

### 2.2. Increasing T and B Lymphocyte Proliferation

Astragaloside has been reported to effectively inhibit cell proliferation in T-cell lymphoma and promote TNFAIP3 expression to inhibit Raji cell proliferation in human B-cell lymphoma while promoting apoptosis [9]. In addition, Treg cells and Th17 cells are subpopulations of helper T cells (Th cells), which are key players in maintaining the physiological balance between immune defense and tolerance. Treg cells primarily mediate peripheral immune tolerance. These cells secrete factors such as IL-10 and transforming growth factor (TGF)-β, which prevent the Th1/Th17-induced immune and inflammatory responses, thereby exerting an immunosuppressive effect [10]. Th17 cells promote inflammation mainly by secreting cytokines such as IL-6, IL-17, IL-23, granulocyte-macrophage colony-stimulating factor, TGF-β, and microRNAs. Thus, there is a reciprocal relationship between Th17 (which promotes the inflammatory response) and Treg (which inhibits this response). In particular, Th17 and Tregs play opposing roles in various immunoinflammatory diseases, typically in a dynamic equilibrium that is critical for maintaining the body’s immunity [11]. In vitro experiments have disclosed that AS-IV modulates the inflammatory response by regulating the balance of Th17/Treg, and studies have revealed an increase in IL-10 and a significant decrease in IL-6, IL-17, IgE, and TGF-β1 levels [12]. In addition, Liu et al. [13] have reported that AS-IV provides therapeutic benefits in the early stages of acute kidney injury, regulates Th1/Th2 imbalance, reduces renal tubular damage, and plays a vital role in protecting the kidneys.

### 2.3. Inhibiting Neutrophil Adhesion-Associated Molecules

Neutrophils play a central role in the inflammatory response. Hence, inhibiting their infiltration and activation can potentially offer a new approach to achieving neuroprotective effects. The chemokine receptor CXCR2 is crucial for the recruitment of neutrophils from circulation to the site of infection. Activation of toll-like receptors in neutrophils downregulates CXCR2 expression and impairs neutrophil migration. AS-IV blocks the reduction in CXCR2 expression and neutrophil migration induced by the TLR4 activator LPS [14]. AS-IV can induce host antimicrobial immunity by modulating GRK2–CXCR2 signaling in neutrophils. CD11b/CD18 is a vital integrin present on the surface of neutrophils that recognizes and firmly adheres to immunoglobulin superfamily proteins on endothelial cells [15,16]. ICAM-1 is a receptor for CD11b/CD18 and induces the aggregation of CD11b/CD18-expressing neutrophils [17]. AS-IV (20 mg/kg, 90 min) has been shown to considerably reduce the level of myeloperoxidase in endothelial cells and prevent neutrophil accumulation in the brain parenchyma. In addition, AS-IV substantially reduces neutrophil adhesion and endothelial cell infiltration by downregulating the expressions of CD11b/CD18 and ICAM-1 in endothelial cells after CIRI [18].

## 3. Antioxidative Effects

### 3.1. Antioxidative Stress

The body produces trace amounts of reactive oxygen species (ROS). Excess accumulation of ROS, or reduced antioxidant capacity, leads to an imbalance in the oxidative and antioxidant systems of the body. These changes result in oxidative stress, which in turn causes inflammatory neutrophil infiltration [19], increased protease secretion [20], and oxidative intermediate generation. Oxidative stress refers to the detrimental effect of free radicals on the body. Excess ROS accumulation leads to impaired mitochondrial function [21], thereby causing dysfunction in cells, tissues, organs, and systems and potentially leading to cancer, atherosclerosis, Alzheimer’s disease, and numerous other diseases. ROS damage mitochondria by triggering mutations in mitochondrial genes, damaging the mitochondrial respiratory chain, disrupting calcium homeostasis, or attacking mitochondrial defense systems [22]. AS-IV is chiefly used to delay or suppress cellular oxidation by scavenging ROS or as an exogenous antioxidant to maintain or reestablish oxidoreductive homeostasis [23].

### 3.2. AS-IV Scavenges ROS and Alleviates Cellular Scorching

Caspase molecules are a group of protease-like molecules that are highly conserved evolutionarily [24]. Natural and synthetic caspase inhibitors can considerably reduce or even block apoptosis induced by various stimuli, and some caspase-knockout animal models exhibit an absence of apoptosis [25]. Furthermore, activated caspase-1 cleaves IL-1β and IL-18 precursors to form active IL-1β and IL-18. Proinflammatory cytokines, such as activated forms of IL-1β and IL-18, are extracellularly released through pores, which leads to the onset of inflammatory responses. Gasdermin D (GSDMD) is a 242-amino acid protein expressed predominantly on the surfaces of the immune and epithelial cells of the small intestinal mucosa [26]. GSDMD has an N-terminal effector and a C-terminal inhibitory structural domain, with the former being the primary functional structural domain involved in cell death via cellular scorching and the latter serving as an autoinhibitor [27]. In vivo, AS-IV, as an exogenous antioxidant, reduces myocardial infarction-induced myocardial fibrosis and cardiac remodeling by inhibiting the ROS/caspase-1/GSDMD signaling pathway [28]. Bone marrow-derived macrophages (BMDMs) are commonly used as primary macrophages for developing inflammatory cell models. In vitro, AS-IV stimulates BMDMs, which possibly alleviates the cardioprotective effect of AS-IV on macrophage death.

### 3.3. AS-IV Regulates Mitochondrial Gene Mutations

Peroxisome proliferator-activated receptor (PPAR) is a member of the intranuclear receptor superfamily of transcription factors that regulate the expression of target genes. PPARγ influences NF-κB signaling, signal transcription, and protein-1-mediated signaling pathway activation [29,30]. These pathways can be suppressed by inhibiting the target gene promoter and transcription. FOXO1 maintains the normal response of β cells to acute oxygen stress, which protects them from oxidative damage by the FoxO protein [31]. Furthermore, AS-IV inhibits oxidative stress in DN by activating the PPARγ–Klotho–FoxO1 axis [32].

Oxidative stress due to ROS is a key activating factor of apoptosis, and superoxide dismutase is one of the main free radical–scavenging enzymes in the body [33]. ROS production, inflammation, and endoplasmic reticulum stress induce podocyte apoptosis. Hyperglycemia, a common stimulus in DN; moreover, inhibits podocyte adhesive function and increases ROS production in mast cells (MCs) in vivo and in vitro. Against this background, the antioxidant and antiapoptotic effects of AS-IV were tested on podocytes under diabetic conditions. The results revealed that AS-IV improved podocyte apoptosis, cysteine-3 activation, and oxidative stress. In addition, AS-IV pretreatment partially restored the mRNA and protein expressions of the apoptosis-promoting gene *Bax* and the apoptosis-inhibiting gene *Bcl-2*. These findings indicate the potential of AS-IV as a novel antioxidant.

### 3.4. AS-IV Regulates Calcium Homeostasis

**Figure 3:**
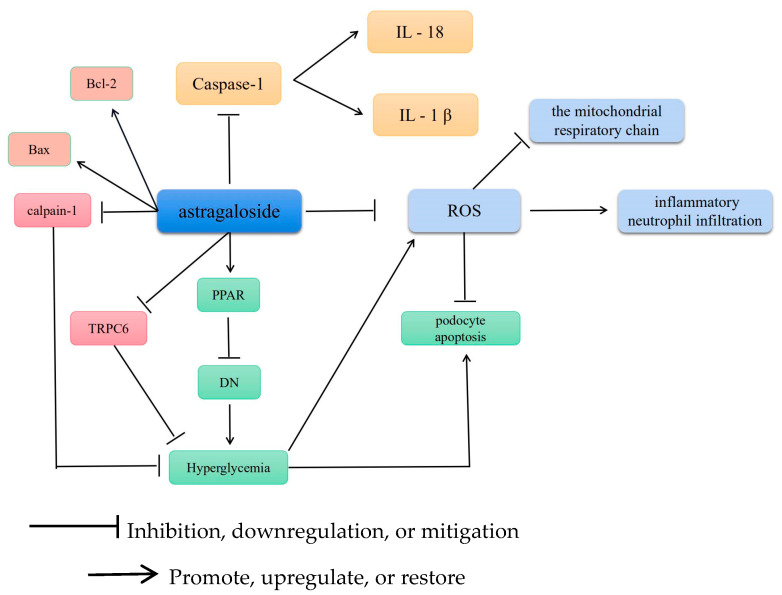
The antioxidative mechanism of AS-IV. Annotation: Bcl-2: B-cell lymphoma-2; Bax: BCL2-Associated X; Caspase-1: cysteinyl aspartate specific proteinase-1; TRPC6: Transient receptor potential cation channel 6; PPAR: Peroxisome proliferator-activated receptor; DN: Deoyribonucleic acid; IL-18: Interleukin 18; IL-1β: Interleukin 1β; ROS: Reactive oxygen species.

AS-IV modulates calcium homeostasis to mitigate cellular oxidation. In a study, diabetic rats were reported to exhibit significantly increased ROS levels and decreased superoxide dismutase and glutathione–Px activities; however, AS-IV administration reversed these changes in a concentration-dependent manner [34]. Particularly, AS-IV prevented hyperglycemia-induced vascular endothelial dysfunction by inhibiting oxidative stress and calpain-1 activation. RPC6, a critical ion channel, is expressed in the kidney, mainly in podocytes. Reportedly, TRPC6 present in podocytes is central to cell signaling regulation in podocytes for calcium homeostasis. AS-IV may prevent hyperglycemia-induced podocyte apoptosis by downregulating TRPC6, which may be mediated by the calcium-regulated phosphatase/NFAT signaling pathway [35]. Therefore, AS-IV could serve as a potential therapeutic agent for diabetic neuropathy. The antioxidant mechanism of AS-IV is illustrated in Figure 3.

## 4. Neuroprotective Effects

Following intravenous AS-IV administration, AS-IV is rapidly absorbed and widely distributed in the liver, kidneys, lungs, heart, and spleen. However, the distribution of AS-IV in the brain is limited owing to the presence of the blood-brain barrier. AS-IV displays a significant protective effect against ischemia–reperfusion injury; however, the potential underlying mechanisms remain unknown.

### 4.1. Preventing Neuronal Loss

AS-IV has been observed to prevent dopamine neuronal loss and behavioral deficits in a mouse model of Parkinson’s disease (PD) [36]. In addition, AS-IV promotes mitochondrial autophagy and reduces the accumulation of damaged mitochondria and mitochondrial ROS production, thereby contributing to the inhibition of astrocyte senescence. SH-SY5Y cells can transform into neuron-like cells and have been widely exploited in in vitro experimental studies of neurological disorders. AS-IV exhibits considerable neuroprotective effects against MPP^+^-induced SH-SY5Y cell death by inhibiting Bax/Bcl-2-related apoptotic pathways and ROS production [37].

PD is characterized by the persistent and irreversible loss of dopamine neurons and the formation of Lewy bodies in the substantia nigra of the midbrain. Although the exact etiology of this disease is yet to be clarified, the probable mechanisms include impaired energy metabolism, oxidative stress, and abnormal protein accumulation.

### 4.2. Action on Neural Stem Cells

After the injury, neural stem cells are attracted to some factors and cross the blood-brain barrier to accumulate at high levels at the injury site; hence, these cells can be targeted for treating neurological diseases such as PD and Frozen disease. AS-IV counters the radiation-induced senescence of brain cells by regulating the p53-p21 and p16-RB senescence-regulated signaling pathways and JNK-p38 phosphorylation [38,39]. Furthermore, the combination of AS-IV and ginsenoside Rg1 augments the protective effects against cerebral ischemic injury via antiapoptotic and anti-inflammatory effects. The underlying mechanism is probably related to the inhibition of NF-κB and JAK1/STAT1 signaling pathway activation upon cerebral ischemia and the regulation of endoplasmic reticulum stress [38]. These findings signify that AS-IV has potential therapeutic value for neuroprotection.

## 5. Antifibrotic Effects

**Figure 4:**
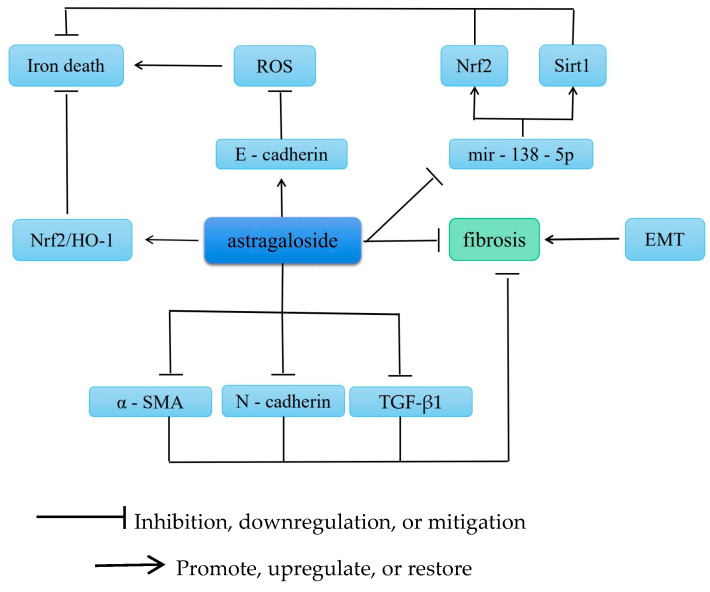
The Anti-fibrotic Mechanism of AS-IV. Annotation: Sirt1: Sirtuin 1; Nrf2: Nuclear factor erythroid2-related factor 2; HO-1: Heme Oxygenase-1; EMT: Epithelial–mesenchymal transition; ROS: Reactive oxygen species; TGF-β1: Transforming growth factor β1; α-SMA: Alpha-smooth muscle actin.

Fibrosis can develop in various organs, and increased fibrous tissue in the organs, decreased parenchymal cells, and excessive deposition of extracellular matrix (ECM) in the tissues are the main pathological changes involved. AS-IV exerts anitpulmonary, anithepatic, antirenal, and antimyocardial fibrotic effects. Furthermore, various growth factors, cytokines, and multiple cell signaling pathways interact and participate in these antifibrotic effects. Figure 4 summarizes the anti-fibrotic mechanism of AS-IV.

### 5.1. Improvement of Renal Fibrosis

Iron death is an inevitable pathological change in end-stage renal disease and is involved in the development of renal fibrosis caused by various diseases. In particular, iron death is a novel form of iron-dependent, nonapoptosis-regulated cell death mainly caused by the reduced biological activity of glutathione peroxidase 4 either because of lipid peroxidation or ROS accumulation [40]. AS-IV prevents iron death in subarachnoid hemorrhage by activating the Nrf2/HO-1 pathway [41]. In a high glucose-induced mouse model, AS-IV has been found to mitigate the effects of high glucose by increasing mir-138-5p expression in retinal pigment epithelial cells and promoting Sirt1 and Nrf2 expression in the nucleus. In addition, AS-IV has been shown to inhibit miR-138-5p expression and enhance Sirt1/Nrf2 activity and cellular antioxidant capacity to mitigate the increase in iron [32]. Epithelial–mesenchymal transition (EMT) is a key process and a direct outcome of renal fibrosis [42]. A study has reported that EMT is associated with decreased epithelial E-cadherin and increased α-smooth muscle actin (α-SMA) expression in mesenchymal cells [43]. In addition, Xu [44] has demonstrated that AS-IV attenuates oxidative stress by inhibiting glycated albumin-induced EMT, alleviating α-SMA expression, and increasing E-cadherin expression in renal proximal tubular cells. Table 1 summarizes the target or pathway of AS-IV in renal fibrosis.

### 5.2. Improvement of Cardiac Fibrosis

AS-IV is effective in preventing and treating cardiovascular diseases, with multiple targets involved in its pharmacological action. The protective effects of AS-IV on the cardiovascular system can be classified into five categories: antimyocardial hypertrophy, anti-ischemia-reperfusion injury, antioxidative stress, Ca^2+^ overload inhibition, and vascular endothelial cell modulation. Multiple mechanisms are involved in the protective effects of AS-IV on the heart. Modulation of cellular signaling pathways, activation or inhibition of related gene expression, and alteration of intracellular calcium ion concentration in cardiac myocytes are a few examples. AS-IV inhibits high glucose-induced oxidative stress and autophagy and protects cardiomyocytes from injury via the miR-34a/Bcl2/(LC3II/LC3I) and pAKT/Bcl2/(LC3II/LC3I) pathways [42]. In addition, AS-IV inhibits apoptosis under various pathological conditions in vivo and in vitro. Moreover, AS-IV has been reported to inhibit the activation of the FAS/FASL signaling pathway in CVB3-induced viral myocarditis (VM) [55]. Therefore, it is evident that astragaloside has a wide range of therapeutic effects for the improvement of cardiac fibrosis. Based on the findings of current studies, it can be used to treat physical injuries caused by hypoxia and ischemia, viral diseases, and novel proangiogenic agents. Table 2 summarizes the target or pathway of AS-IV in cardiac fibrosis.

### 5.3. Improvement of Liver Fibrosis

Hepatic fibrosis is a pathological process in which hepatic stellate cells (HSCs) are activated and proliferate owing to diffuse chronic liver injury caused by various factors, resulting in the excessive synthesis of extracellular collagen and excessive ECM deposition. Fibrosis is a repair response of the body to chronic liver injury. A previous study has shown that liver fibrosis and early cirrhosis are reversible [64]; hence, inhibiting or even reversing the progression of liver fibrosis is crucial for improving the quality of life of patients and the prognosis of liver disease. Rendong et al. [65] have reported that AS-IV exerts an ameliorative effect on CCl4-induced liver fibrosis in rats. A possible mechanism underlying this effect is the downregulation of N-cadherin, α-SMA, and TGF-β1 protein expression along with the upregulation of E-cadherin protein expression. Zhongying et al. [66] have demonstrated that AS-IV inhibits the inflammatory response and plays a vital role in reducing collagen expression by inhibiting the PI3K/Akt/mTOR signaling pathway. The model group exhibited higher levels of α-SMA and TGF-β than the astragaloside group, which suggests that AS-IV significantly improves liver function and liver fibrosis-related protein factors.

Nonalcoholic fatty liver disease (NAFLD) is a major form of chronic liver disease worldwide. AS-IV has been shown to significantly reduce liver tissue damage and serum aspartate transaminase, alanine aminostransferase, and triglyceride levels in NAFLD mice. AS-IV also reduced ROS and malondialdehyde levels and inhibited the LPS-induced production of proinflammatory cytokines (IL-6 and TNF-α) in RAW264.7 cells. Moreover, it downregulated 5-LO and leukotriene B4 expression in NAFLD mice and restored Bax and Bcl-2 expression in PA-treated LO2 cells [67].

Research on primary hepatocellular carcinoma has demonstrated that AS-IV administration delays its development by continuously inhibiting the development of fibrosis via its therapeutic efficacy. Regulation of the pSmad3C/3L and Nrf2/HO-1 pathways, particularly in terms of modulating the reversibility and antagonism of pSmad3C and pSmad3L and promoting Nef2 phosphorylation, is the mechanism involved [66].

## 6. Antitumor Effects

Recently, AS-IV has been reported to exhibit significant antitumor activity [68], indicating its potential as a novel anticancer drug. Protein kinase B (AKT)/endothelial nitric oxide synthase (eNOS), and calpain-1/NF-κB pathways modulate the immune system, regulate noncoding RNA expression, and mediate the sensitivity of anticancer drugs.

### 6.1. Modulation of the Immune System

In vivo studies have demonstrated that AS-IV can enhance the immune response and suppress lung cancer. Within the tumor microenvironment, tumor-associated macrophages (TAMs) are a crucial population of inflammatory cells that can polarize into the M2 phenotype and promote tumor progression. AS-IV exerts its effects by partially inhibiting the M2 polarization of macrophages via the AMPK signaling pathway. This inhibition ultimately leads to a reduction in the growth, invasion, migration, and angiogenesis of lung cancer cells. Therefore, the ability of AS-IV to limit lung cancer metastasis may be attributed to its modulation of TAMs and the immune response [69]. AS-IV has also been found to substantially suppress the migration and invasion of A549 cells by inhibiting the expression of E-cadherin, integrin-β1, MMPs, and Tregs; activating cytotoxic T lymphocytes; and blocking the PKC-α–extracellular regulatory protein kinase (ERK)1/2–NF-κB signaling pathway [70]. In another study, AS-IV administration (50–200 mg/kg, 7 days) clearly increased the proliferation of T and B lymphocytes and antibody production both in vitro and in vivo but inhibited the expressions of IL-1 and TNF-α in peritoneal macrophages in vitro [71].

### 6.2. Control of EMT-Associated Autophagic Pathways for Tumor Suppression

EMT is one of the main features of cellular drug resistance [72]. A study has highlighted the role of AS-IV in the inhibition of EMT because it plays a role in most of the processes associated with AS-IV-related cancers [73]. AS-IV controls several EMT- and autophagy-related pathways, such as phosphoinositol-3-kinase (PI3K)/protein kinase B (AKT), mitogen-activated protein kinase (MAPK)/ERK, Wnt/β-linked protein, and TGF-β/SMAD signaling pathways, which are strongly associated with most tumors. Furthermore, AS-IV has been reported to inhibit EMT and angiogenesis in gastric cancer via miR-195-5p upregulation, demonstrating its potential therapeutic role in gastric cancer via miR-195-5p-regulated PD-L1 [74].

### 6.3. Enhancing Sensitivity to Anticancer Drugs

Tumor multidrug resistance (MDR) is a major cause of chemotherapy failure and cancer recurrence. The drug resistance of tumors can be reversed by blocking the MDR pathway to reduce drug efflux, which can enhance tumor cell chemosensitivity [75]. Qu et al. [76] constructed tumor-bearing mice using Lewis lung cancer cells and treated them with AS-IV. They reported that AS-IV slowed down the proliferation of tumor cells and enhanced cisplatin-induced apoptosis. In addition, AS-IV enhanced taxol-induced apoptosis and G2/M-phase blockade in the cell cycle, thereby enhancing the chemosensitivity of cancer cells to taxol [77,78].

### 6.4. Reduced Integrin-Linked Kinase (ILK)

ILK has emerged as a receptor-proximal protein kinase, a threonine/serine protein kinase that targets adherent spots and is a hub for numerous biochemical signaling pathways in the extracellular and intracellular signaling of integrins [79]. ILK acts in various physiological and pathological processes, such as cell-matrix interaction, cell growth, proliferation, survival, differentiation, tumor metastasis, infiltration, and angiogenesis.

An in vitro study has shown that AS-IV inhibits damage to podocytes owing to high glucose-associated oxidative stress in a dose-dependent manner and reduces the expression of ILK [80]. The antioxidative effect of AS-IV may help protect podocytes by downregulating the expression of ILK [81]. Hence, AS-IV can effectively lower blood glucose levels, reduce urinary albumin excretion, and improve podocyte adhesive function, thereby delaying the progression of DN.

Particularly, ILK expression and activity are upregulated in various tumors and are crucial for tumor diagnosis. Inhibiting ILK activity is an ideal strategy for tumor gene therapy and oncology drugs owing to its ability to induce cell cycle arrest and apoptosis. Therefore, astragaloside AS-IV can serve as a potential agent for tumor gene therapy. Table 3 summarizes the target or pathway of AS-IV in tumors.

## 7. Miscellaneous

Additional unexplored or intensively researched pharmacological effects of AS-IV are of interest, particularly its potential for promoting bone regeneration, improving hair loss, and alleviating constipation.

Both osteoblasts and preosteoclasts contribute to the coupling of osteogenesis and angiogenesis and regulate bone regeneration. According to recent reports, AS-IV accelerates bone regeneration by inhibiting osteoclast formation, preserving preosteoclast cells, and enhancing platelet-derived growth factor-BB (PDGF-BB)-induced angiogenesis. Furthermore, the AKT/GSK-3β/β-catenin signaling pathway regulated by AS-IV may serve as a potential target in distraction osteogenesis (DO) therapy [90]. In addition, iron loading, a common stressor during cellular development, is strongly associated with bone loss and osteoporosis. A study has observed that AS-IV ameliorates the FACC-induced reduction in cell viability, proliferation, pluripotency, and osteogenesis in bone marrow stem cells (BMSCs) [91]. AS-IV has the potential to be developed as a new therapeutic strategy for iron loading-induced aberrant differentiation of BMSCs and osteoporosis. Based on the above-mentioned effect of promoting the repolarization of the macrophage phenotype, which may improve steroid-induced osteonecrosis of the femoral head, AS-IV may exert several effects. Alleviation of osteonecrosis of the femoral head via the repolarization of macrophages from an M1-like phenotype to an M2-like phenotype87 [90], promotion of osteoblasts, alleviation of arthritic symptoms, and reduction of inflammatory cytokines are a few examples.

Apoptosis and premature termination of hair follicle growth are some of the key factors in hair loss. AS-IV has been shown to block the Fas/Fas l-mediated apoptosis pathway and can be used as an alternative treatment for hair loss [92]. However, there are few experiments and related articles regarding its therapeutic effect on hair loss, and this biological activity needs more cellular and animal experiments to prove its reliability and scientific validity.

Intestinal flora and short-chain fatty acids are closely linked to health. Studies have shown that AS-IV can generate butyric acid by regulating the structure of intestinal flora, thereby promoting defecation in slow-transmission constipation mice, improving intestinal motility, inhibiting Cajal cell loss [93], and alleviating colonic lesions. This is one of the major findings of AS-IV in improving constipation. However, as the oral bioavailability of AS-IV is scanty, further experiments are required to prove its reliability and pharmacodynamic evaluation.

## 8. Discussion

Recently, an increasing number of natural products from Chinese herbal medicines, such as chuanxiongzin, danshenone, ginsenoside, and astragalus polysaccharides, have attracted the attention of researchers owing to their diverse and multitargeted effects. AS-IV, which has long been used as a key ingredient derived from astragalus and a nutraceutical, exerts various pharmacological effects on the brain, heart, liver, kidneys, and respiratory system. Anti-inflammatory, antifibrotic, antioxidant, immunomodulatory, and organ-protective effects are exerted via numerous signaling pathways in vital organs and systems. In addition, AS-IV inhibits tumor proliferation and invasion and promotes tumor cell apoptosis in vitro and in vivo. In addition, astragalus has been used for several years for treating diabetes and renal disease owing to its antitumor, cerebroprotective, and multiorgan antifibrotic effects and its ability to alleviate postischemic reperfusion injury in the heart. AS-IV has also been exploited for treating chronic renal disease characterized by abnormal ECM accumulation.

The continuous exploration and validation of the biological activities of AS-IV provide a strong theoretical basis for its development into a clinical drug. However, there are several gaps in comprehending the specific metabolic processes, metabolites, pharmacokinetics, and pharmacodynamics of AS-IV in vivo. In subsequent structural modifications, it is important to consider the ADME (absorption, distribution, metabolism, and excretion) properties of triterpenes. AS-IV, being a tetracyclic triterpene saponin in the form of lanolin ester alcohol, can undergo modifications predominantly in the side chain of the A-ring at the C-2 and C-3 positions and the D-ring. Common modifications include the introduction of hydroxyl, ester, and nitrogen-containing groups. The unique backbone of natural triterpenoids and their numerous modification sites make them highly promising in various fields. For example, triterpenoids have been shown to possess efficient anticancer activity and demonstrate biosafety when used as building blocks in structural assembly. Recently, the emergence of computer-aided drug design and synthetic biology has further expanded research on triterpenoids. These advancements offer new opportunities for studying and modifying triterpenoid compounds to enhance their therapeutic potential.

Indeed, researchers have made considerable progress in unraveling the mechanisms of action of AS-IV in various diseases. However, certain pharmacological mechanisms have not been completely elucidated, such as its effects on respiratory diseases, wound healing, hair loss prevention, constipation relief, and bone growth acceleration. These mechanisms represent potential therapeutic targets for AS-IV. However, the in vivo solubility and bioavailability of AS-IV after oral administration are not very satisfactory. Some researchers have obtained a new water-soluble derivative of AS-IV-astragaloside (LS-102) with higher bioavailability than the parent compound. LS-102 may be a potential agent for the clinical treatment of obesity and related metabolic diseases. However, the metabolic mechanism by which AS-IV acts within the cells needs to be evaluated to understand its uptake, distribution, transformation, and excretion.

## Figures and Tables

**Table 1:** Target or pathway of AS-IV in renal fibrosis.

Research Subject	Induction Methods	Mechanism	Ref.
	T2DM	Alleviates renal tubular epithelial–mesenchymal transdifferentiation through the CX3CL1-RAF/MEK/ERK signaling pathway	[45]
Male diabetes nephropathy rats and	High-fat diet consisting of 8% lard, 10% yolk powder, 18% sucrose, and 0.5% sodium cholate	Downregulation of CD36 expression mediates FFA uptake and lipid accumulation	[46]
diabetic nephropathy rats	using streptozotocin administration in vivo	Inhibiting the excessive proliferation of HG-induced RMCs decreased TGF-β1, Smad3, col1, α-SMA mRNA and protein expression, and increased Smad7 mRNA and protein expression in vitro and in vivo	[47]
Male C57BL/6 mice with renal fibrosis	Unilateral ureteral occlusion (UUO)	Inhibition of TGF-β1 induced EMT	[48]
diabetic KK-Ay mice	Feeding KK-Ay mice a high-fat diet	Inducing autophagy and inhibiting MC activation through the SIRT1-NF-κB pathway	[49]
Male C57BL/6 mice with diabetes	Streptozotocin-induced	Inhibition of the activation of the MEK1/2ERK1/2-RSK2 signaling pathway	[50]
UUO mice	Unilateral ureteral obstruction	Inhibiting inflammation via the TLR4/NF-κB signaling pathway	[51]
Primary renal fibroblasts of BALB/c mice	Treated with TGF-b1	Inhibition of the C and NF-κB signaling pathways	[52]
Male Sprague–Dawley rats with renal fibrosis	Unilateral ureteral obstruction in vivo and TGF-b1-stimulated	Inhibition of TGF-b1, CTGF, a-SMA, and collagen matrix expression, decrease in serum creatinine and urea nitrogen, and upregulation of Smad7, thereby blocking upregulation of TGF-b1, CTGF, and a-SMA, and activation of phosphorylated-Smad2/3	[53]
Male SPF Wistar rats with unilateral ureteral obstruction	Unilateral ureteral obstruction	Inhibition of tubular epithelial–mesenchymal transdifferentiation, fibroblast activation, and an increase in NO production in the kidney	[54]

Annotation: T2DM: Type 2 Diabetes Mellitus; CD36: Platelet glycoprotein 4; TGF-β1: Transforming growth factor beta 1; EMT: Epithelial-mesenchymal transition; MEK1: Mitogen-activated proteinkinase kinase 1; TLR4: Toll-like receptor 4; CTGF: Connective tissue growth factor.

**Table 2:** Target or pathway of AS-IV in cardiac fibrosis.

Research Subject	Induction Methods	Mechanism	Ref.
Male C57BL-6J mice with cardiac fibrosis	Isoprenaline	Increase of Akkermansia, Defluviitaleaceae_UCG-011, and Rikenella abundance and modulation of amino acid metabolism	[56]
Diabetic rats	High glucose/high fat and hypoxia culture condition	Prevented apoptosis and restored cardiac function in MI	[57]
Sprague–Dawley male rats with cardiomyopathy	Adriamycin	Suppressed oxidative stress to counter type I and III collagens, TGF-β, NOX2, and NOX4 expression, and SMAD2/3 activity in the left ventricles	[58]
Cardiac fibrosis rats	Isoprenaline	Inhibited cardiac fibrosis by targeting the miR-135a-TRPM7-TGF-β/Smads pathway	[59]
Male BALB/c mice with cardiac fibrosis	Isoprenaline	Inhibition of the NLRP3 inflammasome pathway	[59]
Cardiac fibrosis rats	Isoprenaline	Inhibited hypoxia-induced cardiac fibrosis in vivo and in vitro is associated with reduced expression of TRPM7	[60]
Male healthy Sprague-Dawley rats with cardiac fibroblast	Isoprenaline	Inhibited ISO-induced cardiac fibrosis proliferation and collagen production through negative regulation of ROS-mediated CT-1 upregulation	[61]
Sprague–Dawley rat pups (age, 1–3 days; weight, 7 ± 2 g)	Isoprenaline	Inhibited ISO-induced cardiac fibrosis by suppressing ROS-mediated MAPK activation	[62]
CVB3-induced inbred male BALB/c mice	CVB3	Downregulated TGF-β1-Smad signaling	[63]
Acute viral myocarditis BALB/c mice	CVB3	Downregulated TGF-β1-Smad signaling	[63]

Annotation: NOX2: NADPH oxidase2; NLRP3 Nucleotide- binding oligomerization domain, leucine- rich repeat, and pyrin domain- containing 3; TRPM7: Transient receptor potential melastatin 7; MAPK: Mitogen-activated protein kinases.

**Table 3:** Target or pathway of AS-IV in tumors.

Research Subject	Induction Methods	Mechanism	Ref.
Primary liver cancer mice	DEN/CCl4/C2H5OH (DCC)	Regulates reversibility and antagonism of pSmad3C and pSmad3L and promotes the phosphorylation of Nrf2	[66]
Male Wistar rats with bile duct ligated	UUO	Induced accumulation of Nrf2 in the nucleus, synthesized antioxidant enzymes through negative regulation of glycogen synthase kinase-3β, scavenged reactive oxygen species, and suppressed hepatic stellate cell activation in bile duct-ligated rats	[82]
HSC rat line HSC-T6	Platelet-derived growth factor (PDGF) family	Promoted cellular senescence and apoptosis by activating the NF-κB pathway to suppress PDGF-BB-induced HSC-T6 activation	[83]
Liver fibrosis mice	Administered carbon tetrachloride (CCl4) to rats	Inhibition of HSC activation and modulation of the TGF-𝛽1/Smad signaling pathway	[84]
Hepatic stellate cells of rats	CCL4	Inhibition of HSC activation and modulation of the TGF-𝛽1/Smad signaling pathway	[84]
Liver fibrosis C57BL/6 mice	Injection with DMN	Decreased collagen deposition, hydroxyproline content, and α-SMA expression levels in the liver tissues	[85]
Diabetic-CCL4 rats	CCL4	Inhibited PAR2 signaling expression	[86]
Male Sprague–Dawley) rats with Cholestatic liver fibrosis	Common bile duct ligation (BDL)	Inhibition of the Notch signaling pathway, thereby inhibiting the abnormal proliferation of biliary epithelial cells	[87]
Hepatic stellate cells of normal male Sprague–Dawley rats	Sequential Pronase and collagenase perfusion	Inhibits HSC activation by inhibiting the generation of oxidative stress and associated p38 MAPK activation	[88]
Hepatic stellate cells of rats	Porcine serum	Inhibitory effects on collagen synthesis and proliferation in HSCs	[89]
